# The evolution of and challenges in defining the clinical endpoint in tuberculosis treatment trials with non-inferiority designs

**DOI:** 10.1186/1745-6215-12-S1-A31

**Published:** 2011-12-13

**Authors:** Patrick PJ Phillips, Angela Crook, Andrew J Nunn

**Affiliations:** 1 MRC Clinical Trials Unit, London, WC2B 6NH, UK

## Background

The first clinical trial to evaluate an effective drug for the treatment of tuberculosis (TB) conducted in 1946 by the Medical Research Council (MRC) under the direction of Bradford Hill is also widely regarded as the first properly conducted randomised clinical trial in any disease area. This new treatment was shown to significantly reduce the short term risk of death in patients with TB. As treatment improved, death became an increasingly uncommon outcome and change in clinical symptoms, radiographic appearance and bacteriological response were the commonly reported endpoints. Eventually, bacteriological failure and relapse became the primary endpoints of interest. Patients lost to follow-up were excluded from the primary analysis, as were patients not completing treatment or changing treatment for adverse events in what would today be regarded as a *per protocol* (PP) analysis.

In recent years the first phase III trials for new TB regimens for several decades have been initiated. Due to the high efficacy of the standard regimen in trial settings, these trials are designed as non-inferiority trials. Guidelines recommend both an intention-to-treat (ITT) and a PP analysis for non-inferiority trials. Classifying all patients with missing endpoints as unfavourable, as is recommended by the US FDA for other infectious diseases, is not appropriate in the context where losses to follow-up in the 12-18 months after treatment (when signs and symptoms have usually ceased) could easily exceed the true bacteriological unfavourable outcome (approximately 5%).

## Objective

In this paper, we will discuss the complexities in (1) defining the composition of the clinical endpoint used in a TB treatment trial with a non-inferiority design and (2) performing the appropriate analyses. The relative roles of bacteriology and clinical signs and symptoms as well as the classification of patients with missing endpoints are important issues which can radically affect the overall trial results.

## Case study

We will illustrate the impact of classifying unassessable patients and unrelated mortality in different ways from recent trials including a regulatory submission to the FDA.

## Conclusions

There is no conservative or ‘best’ approach to defining the endpoints for non-inferiority TB treatment trials and considerable care needs to be taken in formulating a precise definition. Both ITT and PP and other sensitivity analyses are essential to eliminate bias and give robust results. Merely classifying all losses to follow-up as unfavourable leads to meaningless results, since true events will be swamped by random noise.

